# Five Specific Tongue Movements in a Healthy Population

**DOI:** 10.1007/s00455-020-10195-y

**Published:** 2020-10-11

**Authors:** Kilian D. R. Kappert, Simone van Dijk, David Wellenstein, Maarten J. A. van Alphen, Rob J. J. H. van Son, Ludi E. Smeele, Alfons J. M. Balm

**Affiliations:** 1 Department of Head and Neck Oncology and Surgery, The Netherlands Cancer Institute/Antoni Van Leeuwenhoek Hospital, Plesmanlaan 121, 1066 CX Amsterdam, The Netherlands; 2 Robotics and Mechatronics, Technical Medical Centre, University of Twente, Enschede, The Netherlands; 3 Department of Oral and Maxillofacial Surgery, Academic Medical Center, Amsterdam, The Netherlands; 4 Amsterdam Center for Language and Communication, Universtiy of Amsterdam, Amsterdam, The Netherlands

**Keywords:** Tongue cancer, Tongue mobility, Tongue rolling, Tongue folding, Tongue twisting, Cloverleaf

## Abstract

The importance of tongue mobility on speech, oral food transport, and swallowing is well recognized. However, whether the individual tongue mobility influences postoperative function in oral cancer treatment remains to be elucidated. This study assesses the ability to perform five tongue movements as rolling, twisting (two sides), folding, and the ‘cloverleaf’ in a healthy population. Because a tumor in oral cancer patients often restricts the mobility of the tongue, it might be helpful to know if it is possible to recall any of those movements without demonstrating it. Two observers asked 387 Dutch healthy adults if they could perform one of the five specific tongue movements and were subsequently asked to demonstrate the five movements. The distribution in the Dutch population is: rolling: 83.7%, cloverleaf: 14.7%, folding: 27.5%, twisting left: 36.1% and twisting right: 35.6%. The percentage of people that can fold their tongue is almost ten times higher (3% versus 27.5%) than in previous research, and it was found that the ability to roll the tongue is not a prerequisite for folding of the tongue. A relationship between gender or right-handedness and the ability to perform certain tongue movements could not be found. Of the participants, 9.9% and 13.1% incorrectly assumed that they could demonstrate tongue rolling and cloverleaf. Tongue folding and twisting (left or right) were incorrectly assumed in 36.9%, 24.1%, and 25.4% of the cases. Rolling and cloverleaf are preferred for future prediction models because these movements are easy to recall without demonstrating.

## Introduction

The importance of tongue mobility on speech, oral food transport, and swallowing is well recognized. Particularly in oral cancer treatment, the prediction of function loss deserves a lot of attention [1–5]. However, whether the individual tongue mobility influences postoperative function remains to be elucidated [6].

The first article about tongue movements dates back to 1940 and since then, limited data have been published. Six specific tongue movements have been described thus far, being: rolling, folding, twisting (2 sides), cloverleaf, and a pointing tongue [7].

The percentage of people who can roll the tongue varies from 60 to 80% [8–15] and the average percentage of tongue folding lies between 1.5 and 3% [10, 16, 17]. The capability of tongue folding is believed to depend on the presence of the tongue rolling gene [9, 10, 18], but the genetic evidence is doubtful for other movements since little has been published about other features of the tongue, such as the ‘cloverleaf’ tongue [10, 12].

Most of the published data showed no differences for tongue rolling, tongue folding, and the ‘cloverleaf’ tongue between sexes [8, 10, 11, 13, 14, 16]. One study showed a sex difference in the ability to fold and roll the tongue in favor of women [12]. Furthermore, one study showed that right-handed women were able to roll the tongue significantly more compared to right-handed men [15].

**Fig. 1:**
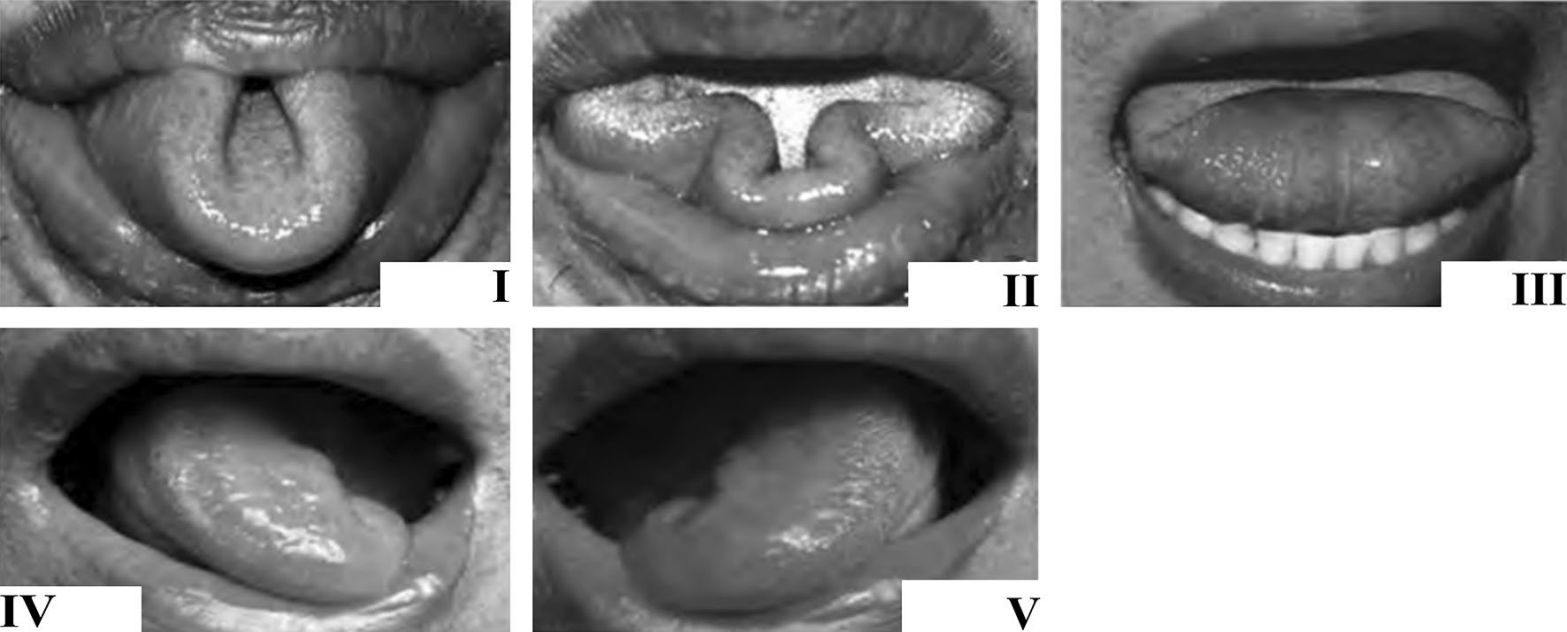
An image with the five specific movements shown to participants just before they are asked about their ability to perform one of the movements. I: rolling, II: cloverleaf, III: folding, IV: twisting left, V: twisting right, folding, III. This image was adapted from Lu (2013) [7]

The main purpose of this study is to assess the ability to perform five of the before mentioned tongue movements as rolling, twisting (two sides), folding, and the ‘cloverleaf’ in a healthy population (Fig. 1). The sixth skill, a pointing tongue, is omitted because the authors thought this movement is too difficult to judge objectively.

Furthermore, we want to investigate if people are aware if they possess the ability to perform any of the specific tongue movements. Some patients do better after partial glossectomy than others, but the reason for this is not always clear.

The muscle topology of the tongue is overall the same among humans, but the innervation is known to differ between individuals [19, 20], which might contribute to the individual versatility of this organ. Pretreatment exercises can influence the postoperative strength and function, and in general, it is assumed that a better preoperative condition also benefits postoperative compensatory movements [21, 22]. It is hypothesized that genetically determined versatility of the tongue could be indicative of the rehabilitation results by the residual mobile capacity. Currently, there’s no literature available suggesting any correlation. However, it is logical to assume that the ability to perform any of the aforementioned specific tongue movements are a derivative of neural control and mobility. Therefore it could be a decisive factor for the prediction of expected postoperative tongue function. Since tongue cancer interferes with the mobility of the tongue, it is a prerequisite that patients can reliably recall if they mastered the task of movement before the tumor occurred.

In summary, the study aims to assess the distribution of five specific tongue movements in a healthy population. Secondly, the awareness of these abilities will be assessed and possible differences in gender and handedness will be evaluated.

## Method

Two observers performed a survey of Dutch healthy volunteers (18 +) recruited at common gathering spots in our institute for four consecutive days. During this period, 387 surveys were conducted, which was enough to reach 95% confidence intervals of less than ± 5% for all movements considering the previous results of LU (2013) [7]. All volunteers were employees of different departments and a variety of functions. Characteristics that were taken into evaluation were gender, native language, and left- or right-handedness. Exclusion criteria were: (history of) a tumor in the oral cavity and any (neuromuscular) condition that might influence the mobility of the tongue. The survey contained two parts. In the first part participants answered two questions I: “Do you know any of the five movements as tongue rolling, folding, twisting, and the ‘cloverleaf’ tongue?”. A picture of the five tongue movements was shown during the question (Fig. 1). Question II: “Do you possess any of the tongue mobility features, and when YES; which ones?”. During the second part, participants showed if they were able to demonstrate the five movements.

Each participant was rated by one of the two raters according to the pictures in Fig. 1. A participant was either scored as capable (1) or as incapable (0) for each of the five tongue movements. Each participant could make three attempts to perform any of the movements. It was allowed to use a mirror during the assessment. When participants accomplished only a part of the movement this was scored as 0: incapable.

Participants were recruited on the institute premises and consisted of employees. All procedures performed in studies involving human participants were in accordance with the ethical standards of the medical ethical committee of the Netherlands Cancer Institute and with the 1964 Helsinki declaration and its later amendments or comparable ethical standards. Written Informed consent was obtained from all individual participants included in the study.

### Statistical Analysis

The Pearson chi-square test was used to assess the correlation between participant characteristics and the ability to perform one of the five tongue movements. The same test was used to assess if there is was a significant difference between the two raters. Because all participants were only rated once, no interrater reliability assessment was performed. For all primary outcome measures the confidence interval for proportions was calculated using the following formula [23]:

$$\hat{P} \pm Z \sqrt {\frac{{\hat{P}\left( {1 - \hat{P}} \right)}}{n}}$$

With: $\hat{P} = \frac{{{\text{No}}\,{\text{of}}\,{\text{cases}} }}{{{\text{Sample}}\,{\text{size}} \left( n \right)}}$ and ${Z= Z}_{\frac{\alpha }{2}}=1.96$ using the *Z*-table (95% confidence interval).

## Results:

### Participant Characteristics

From the 387 participants on whom the survey was performed, 13 were excluded because of incomplete data, resulting in 374 included participants. The mean age was 37 years, ranging from 19 to 64 years old, 61% were females, and 85.8% were right-handed. This survey was performed on employees of the Netherlands Cancer Institute and 92.5% spoke Dutch as their native language.

The Pearson chi-square did not show any association between handedness or gender and the ability to perform a certain movement (*P* > 0.05, Table 1).

**Table 1:** The Pearson chi-square test and significance value for each of the movements vs handedness or gender

	Pearson chi-square		Asymptotic significance (2-sided)	
	Handedness	Gender	Handedness	Gender
I: Rolling	0.894	0.567	0.334	0.753
II: Cloverleaf	0.80	1.018	0.777	0.601
III: Folding	0.18	2.847	0.893	0.241
IV: Twisting (left)	0.10	1.595	0.921	0.451
V: Twisting (right)	0.035	1.688	0.852	0.430

### Agreement Researchers

A chi-square test was performed for the two raters and every type of tongue movement (Table 2). The test showed no association with the observer and the outcome (*P* > 0.05).

**Table 2:** Pearson chi-square test and significance value to test if the difference between raters is significant

	Pearson chi-square	Asymptotic Significance (2-sided)
I: Rolling	2.354	0.125
II: Cloverleaf	3.061	0.080
III: Folding	3.566	0.059
IV: Twisting (left)	0.877	0.349
V: Twisting (Right)	0.079	0.778

Table 3a shows the percentage of the participants that can and think they can perform a certain type of movement. Tongue rolling is the easiest tongue movement which can be done by 83.7% of the participants, and only 9.9% misjudge their ability to do so (Table 3b). The cloverleaf is the hardest movement to carry out; only 14.7% of the participants could do so. The misjudgment for this movement is relatively low, as can be seen in Table 3b. The 95% confidence intervals remained  < ± 5% for all movements. Table 3b also shows conditional probabilities, for example, the percentage from the participants that think they can fold, but cannot (3b, type1). Almost half of the participants think they can make a folding or a left/right twist with their tongue, while the actual success rate is about 8–16% less. The misjudgment (Table 3b) of these three movements varies between 24 and 37%. Folding is the most overestimated movement since 56.3% of the participants (Table 3b, type 1) failed. As a result of smaller subgroups, some combinations of misjudgments of movements have 95% confidence intervals >  ± 5%.

**Table 3:** Percentages and 95% confidence interval of the participants who (a) can/think they can (b) judged their ability wrong by type 1: think they can but actually cannot and type 2: think they cannot but actually can

	I: Rolling	II: Cloverleaf	III: Folding	IV: Twisting (left)	V: Twisting (Right)
(a) Abilities					
Can	83.7% ± 3.7	14.7% ± 3.5	27.5% ± 4.5	36.1% ± 4.9	35.6% ± 4.9
Think	77.0% ± 4.3	5.3% ± 2.28	43.6% ± 5	43.6% ± 5	44.9% ± 5
(b) Wrong judgment	9.9% ± 3.0	13.1% ± 3.4	36.9% ± 4.9	24.1% ± 4.3	25.4% ± 4.4
Type 1: Think yes but cannot	2.1% ± 1.7	35.0% ± 21.0	60.7% ± 7.5	36.4% ± 7.4	38.7% ± 7.4
Type 2: Think not but can	36.0% ± 10.2	11.9% ± 3.4	18.5% ± 5.2	14.7% ± 4.8	14.6% ± 4.8

Only 32.5% of the participants can perform more than two separate movements with their tongue (Table 4). Participants who think they can perform two or three tongue movements overestimate their ability to carry out complex movements by about 8%. Participants who can perform all of the five movements generally underestimate their ability to do so by about 3%. While no correlation could be found between gender and the ability to perform a certain movement, there appears to be a slight advantage for women in the number of movements that can be performed. There are more women than men that can perform 2 and 3 movements. There are slightly more men that can perform all five movements. There is no indication that men overestimate there ability more than women and visa versa.

**Table 4:** Percentages of female, male and total number of participants who can perform and think they can perform at least a number of ‘*x*’ movements

	None (%)	1 or more (%)	2 or more (%)	3 or more (%)	4 or more (%)	5 (%)
Female						
Can	11.1	88.9	59.3	35.4	16.4	4.0
Think	9.7	90.3	67.0	42.3	17.2	1.8
Male						
Can	12.4	87.6	47.6	28.3	16.6	6.2
Think	11.0	89.0	58.2	38.4	21.2	2.1
Total						
Can	11.6	88.4	54.8	32.5	16.4	4.8
Think	10.2	59.8	63.4	40.6	18.7	1.9

Table 5 shows the percentage of participants that can perform a specific movement (column), at the condition that a second movement (row) can be performed. Table 6 shows the percentage of participants that can perform a specific movement (column) at the condition of not being able the perform the second movement (row). When participants can perform one of the more complex movements (II to V) it is more than 90% likely that they can also perform a rolling (I) movement. Participants that can perform the cloverleaf (II) are the best all-round by being able to perform on average more than 60% of all other movements. Also interesting is that 75% of participants that can twist to the left, can also twist their tongue to the right and vice versa. Not possessing the ability to perform a certain movement never excludes the ability to perform one of the others.

**Table 5:** Conditional probability of being able to perform a certain movement, but also another movement: If Row(R), Than Column(C)

*R* → *C*	I: Rolling (%)	II: Cloverleaf (%)	III: Folding (%)	IV: Twisting (left) (%)	V: Twisting (Right) (%)
I: Rolling	**100**	20	33	43	42
II: Cloverleaf	97	**100**	62	66	65%
III: Folding	94	36	**100**	62	61
IV: Twisting (left)	95	30	49	**100**	75
V: Twisting (right)	95	30	49	76	**100**

**Table 6:** Conditional probability of not being able to perform a certain movement, but being able to perform another movement: If not Row(R), Than Column(C)

~ *R* → *C*	I: Rolling (%)	II: Cloverleaf (%)	III: Folding (%)	IV: Twisting (left) (%)	V: Twisting (Right) (%)
I: Rolling	**0**	3	11	13	13
II: Cloverleaf	82	**0**	23	32	32
III: Folding	80	10	**0**	28%	28
IV: Twisting (left)	78	10	18	**0**	15
V: Twisting (right)	78	10	19	15	**0**

## Discussion

To our knowledge, we are the first to study the distribution of distinctive tongue movements and the awareness thereof in a large population. Rolling the tongue was the movement that could be performed by most people (83.7%). The cloverleaf seemed to be the hardest movement and could only be performed by 13.7%. The correct judgment of the ability to perform a tongue movement ranges between 63.1 and 90.1%. A relationship between gender or right-handedness [15] and the ability to perform certain tongue movements, as in other studies, could not be found [8, 10–14, 16]. There were however slightly more women that could perform a total of 3 or 4 movements.

## Literature Comparison

According to other literature, 60% to 80% of the population can roll their tongue [8–15]. In our study, this number is even higher; 83.7% (Table 3a). The percentage of people being able to fold (III) their tongue is much higher in our sample (27.5%) than in previous research (1.5% to 3%) [9, 10, 17]. While the folding movement can be difficult to determine, as there are different levels of folding which could potentially be judged differently, it is still an exceptionally high difference. This might indicate regional differences for this type of movement as the aforementioned research was conducted in China, India, and the United States [9, 10, 17]. Whether tongue training affects mobility is not clear. Hirschhorn [24] investigated different tongue features in a single family and found that two family members of a study population of thirteen, were able to learn tongue folding within two weeks of training. The evidence, however, is low because of the small study population. Kothari et al. [25] showed that one hour of tongue training results in an improvement of tongue strength, which is not influenced by the ability of tongue rolling.

Since others found that tongue folding might also depend on practice, it could as well be related to the language [24, 25]. There is, however, not enough material to confirm such a theory. Being able to twist the tongue to one side doesn’t necessarily imply that someone can twist to the contralateral side since only 75% of the people who can twist the tongue can do this bilaterally (Table 5).

The ability to roll the tongue is believed to be caused by a dominant gene, whereas the ability to fold the tongue is believed to be of a recessive character [11, 16, 17]. Some reports state that the ability to fold the tongue depends on the rolling tongue gene [9, 10, 18]. In this study, these results are not observed, as 11% of the people that can fold the tongue cannot roll their tongue (Table 6). This is strengthened by studies among monozygotic and dizygotic twins that suggest that tongue rolling is not entirely genetically determined [13, 26, 27]. Around 95% of people that can perform a complex movement other than rolling will also be able to roll their tongue. It seems that this movement is often a prerequisite to perform other movements.

While the shape and size of the tongue differ greatly between persons, the muscular arrangement in humans seems to follow a strict pattern [19, 20]. However when looking at motor innervation, a different picture arises. The hypoglossal nerve has two different types of possible topologies: single branching (40%) and multiple branching (60%) [19]. However, previous research from our group shows that a specific global branching topology is not limited to characteristic muscle activity. Even within one patient, both sides of the tongue can have different distal branching topologies resulting in different muscle activations on a micro level [28]. The variety in distal branching patterns might be the reason that there are so many differences between the tongue abilities of people. Also, this study showed that of the subjects who can perform a tongue twist, not everybody can perform a twist to both the left and the right side, confirming that there is a difference in lateral innervation (Table 5). This could as well be one of the core concepts explaining why patients with the same tumor characteristics evolve different speech and swallowing problems [4].

## Judgment Regarding Own Abilities

Whether there is a relationship between the ability to perform complex tongue movements and postoperative tongue function remains to be investigated in future research. However, we do know that when a tumor restricts the mobility of the tongue, specific tongue abilities might be lost. Therefore, when conducting a study into this matter it is essential to know if people can judge their tongue mobility before demonstrating.

The majority (83.7%) of the population was able to roll the tongue (I) and only 14.7% were able to fold the tongue (Table 3a). Both abilities are slightly underestimated by the population, but the “wrong judgment” is very low (9.9% and 13.1%). The low level of wrong judgments makes these abilities potentially useful to distinguish patients in postoperative functionality. However, since the ability for rolling is relatively common and folding relatively uncommon, there is less room to differentiate between groups. The group that can roll, but not a cloverleaf is by far the largest.

About one-third of the population can perform a folding (III) movement or a twisting (left/right) movement. This 1/3 ratio is more ideal for decision making since the subdivided groups have more balanced sizes. These abilities are, however, often overestimated. The wrong judgment, which ranges from 24.1 to 36.9%, largely consists of people who think they can perform this movement, but cannot. With these movements, the physician can only be confident in 63.1% to 73.9% of the cases that the patient can make a proper judgment about their ability.

By looking at the percentage of wrong judgment, tongue rolling and cloverleaf seem to be the best abilities to test for a postoperative prediction model. However, discrepancies between the specific tongue movements are large. Cloverleaf performance is found in 14.7%, whereas tongue rolling is present in 83.7%. This means that there are a large number of people, who can perform rolling but cannot perform cloverleaf. These observations limit the number of patients that could benefit from the model and pose a potential problem for future study designs. Nevertheless, we are convinced that knowledge of the effect of preoperative tongue movements on postoperative mobility can be used to gain more insight into the mechanisms that influence the success rate of rehabilitation post-surgery.

## Limitations

Both observers discussed the scoring of different movements beforehand. However, judging whether someone can perform a movement can be a matter of interpretation. It appeared, that mainly small differences emerge due to different interpretations by the observer despite the use of clear images. The chi-square test and manual check of the frequencies confirm that no significant incongruencies occurred. Limited data about tongue movements have been published since the first article in 1940. Neither are demonstration materials other than images available. Therefore it was decided to use the five instructive images from a previous paper on this subject [7]. Yet it is unknown if, for example, video instructions would result in different performances by the participants.

Because all participants were recruited on institute premises, it is questionable if it does represent the Dutch population in all its aspects. It would, for example, be interesting to subdivide groups in different dialects spoken in the Netherlands or based on ethnicity. Nevertheless, this topic was out of the scope of this study.

## Future Perspectives

Knowing if, and how many possess the ability to perform any of the specific tongue movement is a prerequisite for further research towards predicting the functional outcome. Future research could focus on assessing if there is a difference in functional outcome after treatment between the patients that can roll the tongue, can make a cloverleaf, both or neither of these. This potential prediction model can be used in combination with or instead of more advanced techniques such as biomechanical modeling or optical tracking. These options might take longer to implement and validate in a clinical setting [29, 30]. Also when the genetics and physiology behind the ability to perform different features can be clarified, it might increase insights in the prediction of postoperative reduced tongue mobility or may be helpful in oral rehabilitation.

## Conclusion

Tongue movement features and its distribution in population have an unknown physiological background and have not received much scientific attention yet. This study assessed the distribution of five specific tongue movements in the Dutch population: rolling: 83.7%, cloverleaf: 14.7%, folding: 27.5%, twisting left: 36.1%, and twisting right: 35.6%. An additional finding is that the percentage of people that can fold their tongue is almost ten times higher (3% versus 27.5%) than in previous research. It was found that the ability to roll the tongue is not a prerequisite for folding of the tongue.

In this study, participants were most aware of their ability to roll the tongue and to make a cloverleaf. While the group size difference between people who can and cannot do these movements is relatively large, rolling and cloverleaf are still preferred over folding and twisting (left and right) for future prediction models, based on the low number of misjudgments with these movements.
